# Nonoperative Management of a Major Traumatic Hepatic Injury in a Five-Year-Old Boy

**DOI:** 10.7759/cureus.93501

**Published:** 2025-09-29

**Authors:** Saud Aljadaan, Meral Alzimam, Zahra AlMatar, Abdulaziz A Alkoblan

**Affiliations:** 1 Pediatric Surgery, King Abdulaziz Medical City, Riyadh, SAU; 2 Pediatric Surgery, King Faisal Specialist Hospital and Research Centre, Riyadh, SAU; 3 Pediatric Surgery, King Saud Medical City, Riyadh, SAU

**Keywords:** abdominal trauma in children, liver injury, nonoperative management, pediatric trauma, traumatic hepatic injury

## Abstract

Nonoperative management (NOM) is the standard approach for stable pediatric liver trauma, though rare complications like bile leaks may occur. We present the case of a five-year-old boy with a grade V liver laceration following blunt abdominal trauma. Despite initial hypotension, he was stabilized and managed nonoperatively. On day 5, he developed jaundice and abdominal distension due to a significant bile leak. Imaging confirmed biliary injury, and the leak was successfully managed with percutaneous drainage and endoscopic sphincterotomy with common bile duct stenting. This case highlights the effectiveness of minimally invasive interventions in managing traumatic bile leaks during NOM.

## Introduction

Nonoperative management (NOM) is the standard approach for stable pediatric liver trauma, though rare complications such as bile leaks may occur. The most frequent causes of liver injury in children include motor vehicle accidents, falls, blunt abdominal trauma, and child abuse. Injuries are classified from grade I to grade V or described more broadly as mild, moderate, or severe, with management decisions guided primarily by the patient's hemodynamic status [1].

Bile leak is an uncommon but important complication, typically presenting days after the initial trauma. Clinical features include abdominal pain, progressive distention, and jaundice, resulting from bile leakage into the hepatic parenchyma, peritoneal cavity, or pleural cavity [2].

Here, we report the case of a five-year-old boy with a grade V liver laceration complicated by bile leakage, which was successfully managed nonoperatively.

## Case presentation

In February 2021, a five-year-old boy sustained blunt abdominal trauma after being rolled over twice by a car at low speed. On arrival, he was hypotensive (71/50 mm Hg; heart rate (HR): 140 bpm) with mottled skin and a tense, distended abdomen. Initial resuscitation included two units of packed red blood cells (RBCs) (10 mL/kg each) and two 20 mL/kg normal saline boluses. He was stabilized after four hours without inotropes.

**Figure 1:**
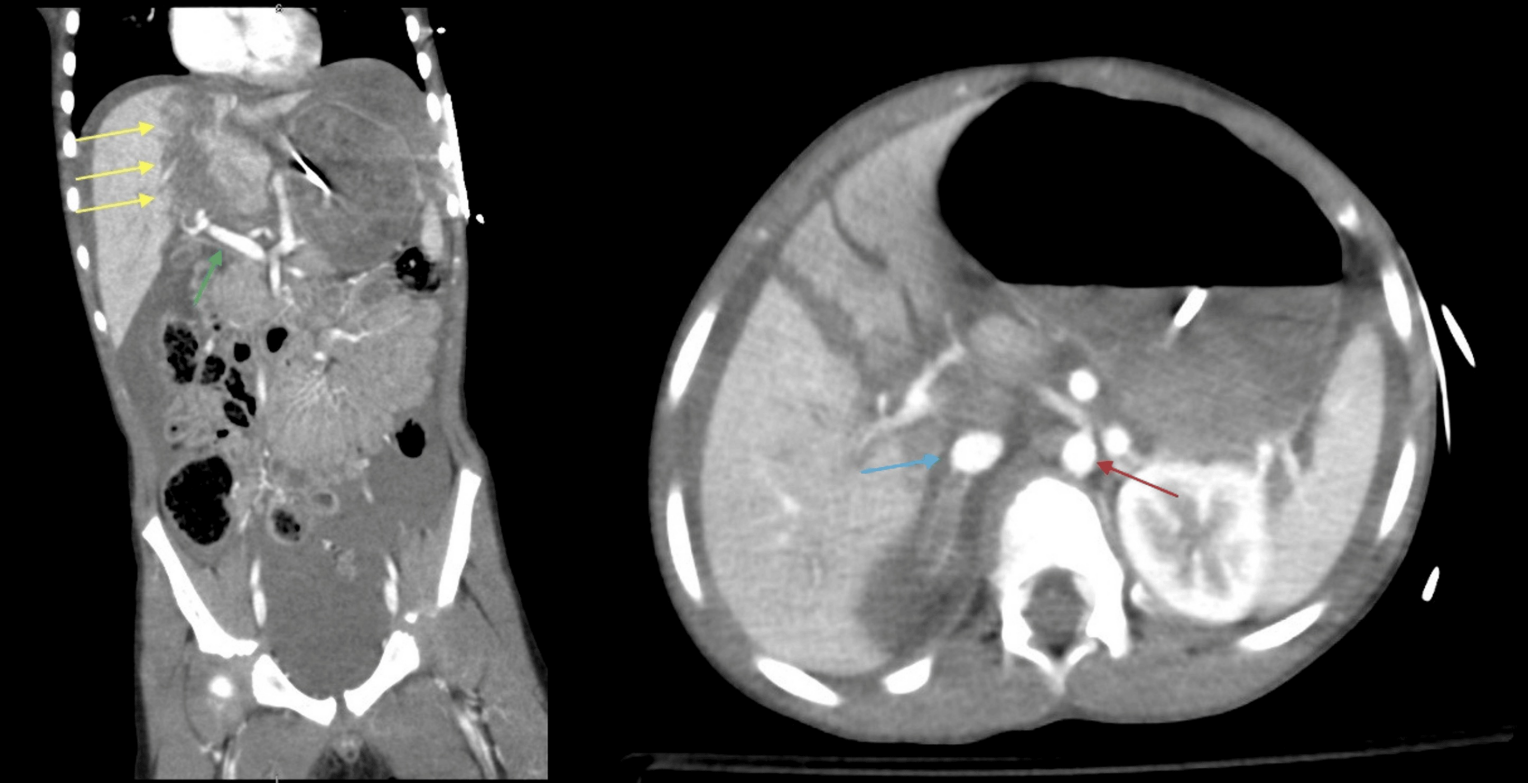
Computed tomography scan showing a high-grade liver injury Red arrow: aorta; blue arrow: inferior vena cava; green arrow: portal vein; yellow arrow: high-grade liver injury

Computed tomography (CT) imaging revealed a grade V right hepatic lobe laceration with vascular injuries involving the inferior vena cava (IVC) and middle hepatic vein, perihepatic hematoma, hemoperitoneum, mild intrahepatic biliary dilation, and right adrenal hematoma (Figure 1). 

**Figure 2:**
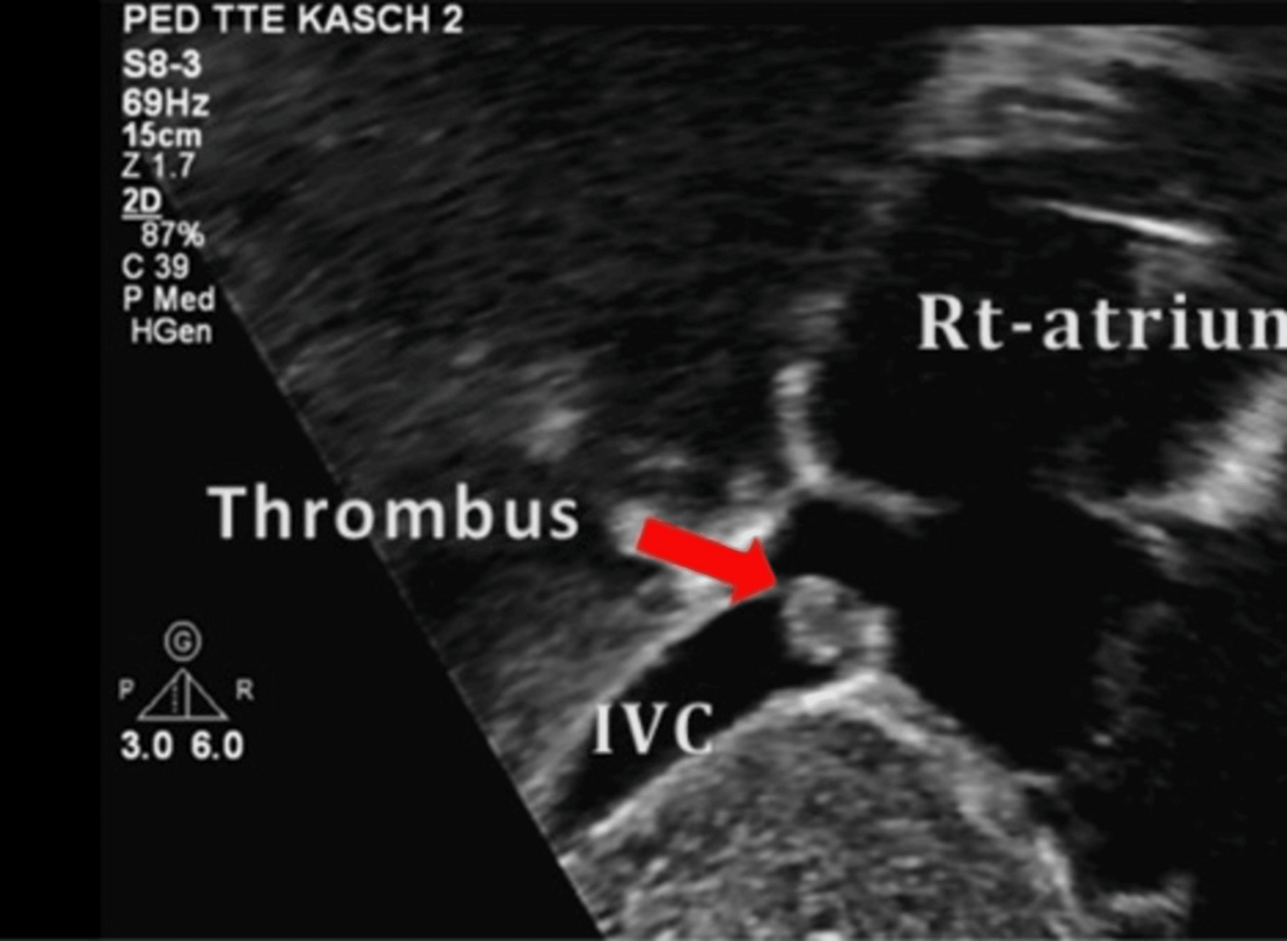
Echocardiography showing a thrombus at the junction of the inferior vena cava and right atrium

The patient remained hemodynamically stable, and as a result, the decision was made to continue with NOM under close observation. An echocardiogram at the time of initial presentation reported a mass at the site of the IVC and right atrial junction, measuring approximately 1.6×1.7 cm, most likely representing a thrombus (Figure 2).

**Figure 3:**
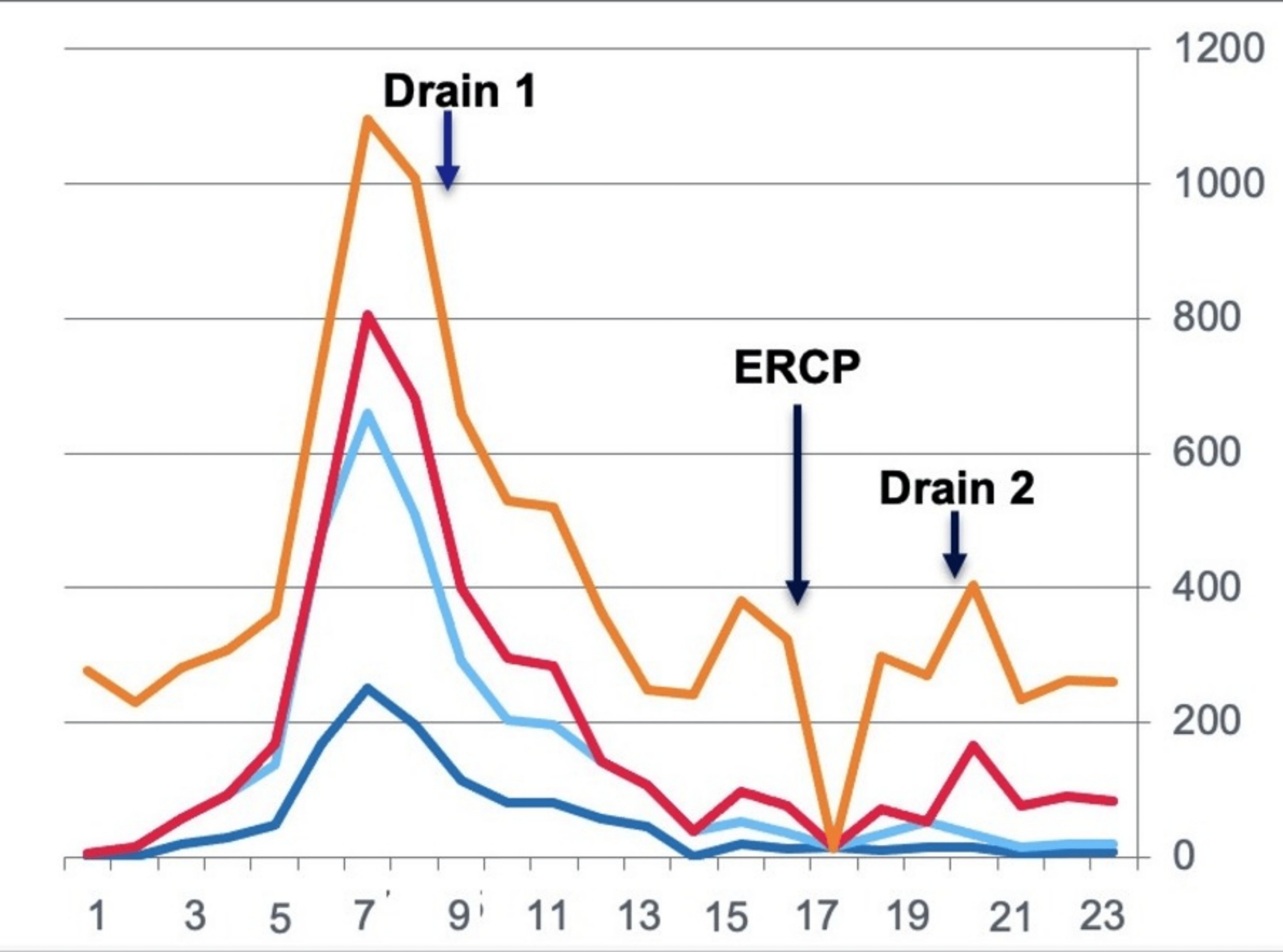
Laboratory investigations during the patient's stay Yellow (alkaline phosphatase): 156-369 U/L; red (gamma-glutamyl transferase): 12-64 U/L; light blue (total bilirubin): ~20.5 umol/L; dark blue (direct bilirubin): ~8.6 umol/L ERCP: endoscopic retrograde cholangiopancreatography

Throughout his treatment, the patient's serial hemoglobin levels remained stable, while his total serum bilirubin levels were trending upward, reaching 409 mg/dL (Figure 3). On day 5 post-trauma, the patient developed jaundice and progressive abdominal distension. Abdominal ultrasonography revealed a large intra-abdominal collection. An ultrasound-guided percutaneous drainage catheter was inserted, draining 1,780 milliliters of bile-stained fluid. Magnetic resonance cholangiopancreatography (MRCP) showed a highly suspicious partial injury to the common hepatic duct and common bile duct. The patient was managed with bowel rest, antibiotics, and total parenteral nutrition.

Ongoing bile drainage prompted endoscopic retrograde cholangiopancreatography (ERCP), which confirmed a leak from the common bile duct. Sphincterotomy and stent placement were performed. Three weeks later, residual septated bilomas were drained percutaneously, and all drains were removed two weeks afterward. The stent was removed 10 weeks post-insertion. No ERCP-related complications occurred.

During hospitalization, the patient developed left thigh deep venous thrombosis (DVT). A hematology consultation was obtained, and anticoagulation was withheld due to high bleeding risk from the perihepatic hematoma. Serial Doppler ultrasound showed spontaneous partial recanalization of the left external iliac vein. The patient gradually resumed oral feeding and was discharged after nearly two months in stable condition. At follow-up, he remained symptom-free with normal imaging and biochemical results.

## Discussion

The success rate of NOM for liver injuries has been reported to exceed 90%, even in cases of high-grade injuries [3-5]. The most concerning complication of NOM is rebleeding, which can result from hematoma or pseudoaneurysm rupture. The incidence of rebleeding ranges from 1.7% to 5.9%, and it carries a mortality rate of 18%. However, studies have shown that 70% of delayed bleeding can be managed conservatively [1,6,7].

The incidence of traumatic biliary injury in pediatric trauma is estimated to be between 0.05% and 4% [2,8,9]. Most of these injuries (74%) occur in grades III-V liver injuries [10]. Traumatic biliary injuries may involve intrahepatic, extrahepatic, or both duct systems, with the right intrahepatic duct being the most common site of injury following blunt abdominal trauma [11-13].

Early diagnosis of bile leaks necessitates a high index of suspicion. Patients often present with increasing abdominal pain, distension, nausea, and vomiting between three and seven days following the initial trauma [8,11]. In our case, symptoms first appeared on the fifth day after the initial injury, which aligns with findings from other series [11].

Most traumatic bilomas resolve spontaneously without intervention; however, percutaneous drainage is indicated for infected or symptomatic bilomas [6,8]. In cases of free intraperitoneal bile leaks, intraperitoneal drainage (IPD) is recommended as the first-line treatment [11]. IPD alleviates symptoms associated with the pressure effects of a bile leak, reduces the chance of superinfection, and converts a free bile leak into a controlled fistula, which may suffice for resolving minor leaks [12-14].

For our patient, once progressive abdominal distension developed, peritoneal drainage was the first line of management. The drain output and color, along with laboratory findings, confirmed the presence of an intraperitoneal bile leak, prompting a referral for MRCP. MRCP, due to its ability to provide both anatomical and functional details of biliary injuries, is superior to hepatobiliary iminodiacetic acid (HIDA) scans in cases with high clinical suspicion [15].

Patients with ongoing biliary leaks after peritoneal drainage, or those with major leaks characterized by large daily volumes of drain output, are often managed with ERCP and endobiliary stent placement [9,11,12]. Stenting and sphincterotomy aim to reduce the transpapillary biliary-duodenal pressure gradient while the duct injury heals [2,13]. However, ERCP carries risks including pancreatitis, bleeding, infection, and perforation, which must be weighed against its benefits [14].

In rare cases of bile peritonitis, laparotomy or laparoscopic irrigation/drainage with endoscopic bile duct stent placement may be required [16]. Most bile injuries resolve within two weeks following ERCP (3-64 days), with drainage catheters typically being removed after a median of 23 days [8]. In our case, the drain was removed after five weeks once the drainage output ceased. Similar studies recommend stent removal between three and eight weeks after insertion [12]. Our patient had the stent removed 10 weeks post-insertion.

Vascular injuries remain a critical consideration in high-grade liver trauma. Although our patient had vascular involvement, including a thrombus at the IVC-right atrial junction, he was managed nonoperatively because of hemodynamic stability and the high morbidity of surgical exploration in this setting [1,4,10]. Surgical intervention is generally reserved for cases with ongoing hemodynamic instability, uncontrolled hemorrhage, or failed NOM [1,4].

Although venous thromboembolism (VTE) is a common cause of morbidity and mortality in adults, the risk of VTE in children under 13 years old is almost negligible, and routine anticoagulation prophylaxis is not recommended [17]. However, on the 12th day of hospitalization, our patient developed DVT in the lower limb. Anticoagulation was withheld due to the high risk of bleeding, as indicated by the patient's international normalized ratio (INR) being near the upper limit. Three weeks later, a repeat Doppler ultrasound showed spontaneous recanalization of the left external iliac vein. The management of VTE in pediatric trauma patients requires careful consideration of the bleeding risk versus the potential for pulmonary embolism, and this should be individualized in each case [18,19].

## Conclusions

This case illustrates that NOM, supplemented with percutaneous drainage and ERCP stenting, can effectively treat bile leaks complicating high-grade pediatric liver trauma. Despite the presence of portal vein thrombosis, withholding anticoagulation was justified due to bleeding risk, underscoring the importance of individualized, context-specific decision-making. The child recovered fully, with the resolution of the bile leak and stable discharge.

This case adds to the growing evidence supporting NOM even in high-grade pediatric liver injuries when managed in a multidisciplinary tertiary care setting, highlighting the importance of close collaboration among pediatric surgeons, intensivists, hematologists, and interventional radiologists to ensure optimal outcomes. It reinforces the role of minimally invasive strategies in addressing biliary complications while drawing attention to the ongoing debate regarding vascular thrombosis in pediatric trauma and the need for clearer evidence-based guidance.
